# Cytenamide acetic acid solvate

**DOI:** 10.1107/S160053680801550X

**Published:** 2008-05-30

**Authors:** Andrea Johnston, Alastair J. Florence, Francesca J. A. Fabianni, Kenneth Shankland, Colin T. Bedford

**Affiliations:** a Solid-State Research Group, Strathclyde Institute of Pharmacy and Biomedical Sciences, John Arbuthnott Building, University of Strathclyde, 27 Taylor Street, Glasgow G4 0NR, Scotland; b University of Göttingen, GZG, Department of Crystallography, Goldschmidtstrasse 1, D-37077 Göttingen, Germany; c ISIS Facility, Rutherford Appleton Laboratory, Chilton, Didcot, Oxon OX11 0QX, England; d University College London, Department of Chemistry, 20 Gordon Street, London WC1H 0AJ, England

## Abstract

In the crystal structure of the title compound (systematic name: 5*H*-dibenzo[*a*,*d*]cyclo­hepta­triene-5-carboxamide ethanoic acid solvate), C_16_H_13_NO·C_2_H_4_O_2_, the cytenamide and solvent mol­ecules form a hydrogen-bonded *R*_2_^2^(8) dimer motif, which is further connected to form a centrosymmetric double ring motif arrangement. The cycloheptene ring adopts a boat conformation and the dihedral angle between the least-squares planes through the two aromatic rings is 54.7 (2)°.

## Related literature

For details on experimental methods used to obtain this form, see: Davis *et al.* (1964 ▶); Florence *et al.* (2003 ▶); Florence, Johnston, Fernandes *et al.* (2006 ▶). For related literature on related mol­ecules, see: Cyr *et al.* (1987 ▶); Fleischman *et al.* (2003 ▶); Florence, Johnston, Price *et al.* (2006 ▶); Florence, Leech *et al.* (2006 ▶); Bandoli *et al.* (1992 ▶); Harrison *et al.* (2006 ▶); Leech *et al.* (2007 ▶); Florence *et al.* (2008 ▶) and Johnston *et al.* (2006 ▶). For other related literature, see: Etter (1990 ▶).
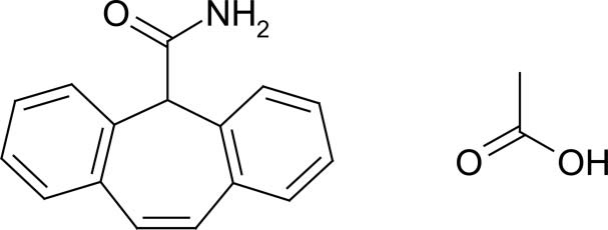


## Experimental

### 

#### Crystal data

C_16_H_13_NO·C_2_H_4_O_2_
*M* *_r_* = 295.34
Monoclinic, 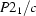

*a* = 5.8726 (17) Å
*b* = 14.418 (3) Å
*c* = 18.182 (4) Å
β = 95.13 (2)°
*V* = 1533.3 (6) Å^3^
*Z* = 4
Mo *K*α radiation
μ = 0.09 mm^−1^
*T* = 160 K
0.44 × 0.09 × 0.06 mm

#### Data collection

Oxford Diffraction Gemini diffractometer
Absorption correction: multi-scan (*ABSPACK*; Oxford Diffraction, 2007 ▶) *T*_min_ = 0.84, *T*_max_ = 0.99
16235 measured reflections
2759 independent reflections
2025 reflections with *I* > 2σ(*I*)
*R*_int_ = 0.065

#### Refinement

*R*[*F* ^2^ > 2σ(*F* ^2^)] = 0.089
*wR*(*F* ^2^) = 0.148
*S* = 1.08
2759 reflections
199 parameters
H-atom parameters constrained
Δρ_max_ = 0.43 e Å^−3^
Δρ_min_ = −0.37 e Å^−3^

### 

Data collection: *CrysAlis CCD* (Oxford Diffraction, 2007 ▶); cell refinement: *CrysAlis CCD*; data reduction: *CrysAlis RED* (Oxford Diffraction, 2007 ▶); program(s) used to solve structure: *SIR92* (Altomare *et al.*, 1994 ▶); program(s) used to refine structure: *CRYSTALS* (Betteridge *et al.*, 2003 ▶); molecular graphics: *PLATON* (Spek, 2003 ▶) and *ORTEP-3* (Farrugia, 1997 ▶); software used to prepare material for publication: *PLATON*.

## Supplementary Material

Crystal structure: contains datablocks I, global. DOI: 10.1107/S160053680801550X/om2234sup1.cif

Structure factors: contains datablocks I. DOI: 10.1107/S160053680801550X/om2234Isup2.hkl

Additional supplementary materials:  crystallographic information; 3D view; checkCIF report

## Figures and Tables

**Table 1:** Hydrogen-bond geometry (Å, °)

*D*—H⋯*A*	*D*—H	H⋯*A*	*D*⋯*A*	*D*—H⋯*A*
N1—H1N⋯O2^i^	0.88	2.27	2.888 (4)	128
N1—H2N⋯O2^ii^	0.88	2.18	3.018 (4)	158
O3—H3⋯O1^iii^	0.84	1.73	2.565 (4)	169

Symmetry codes: (i) 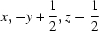; (ii) 
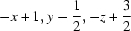
; (iii) 
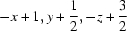
.

**Fig. 1.**
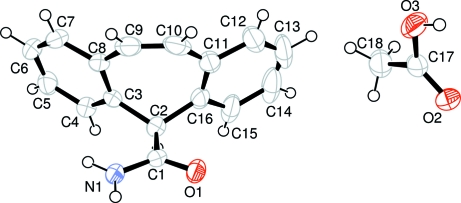
The molecular structure of CYT acetic acid (1/1), showing 50% probablility displacement ellipsoids.

**Fig. 2.**
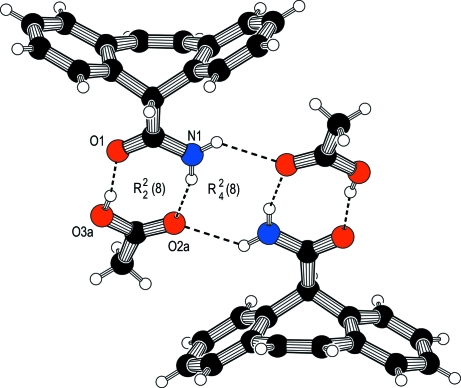
The hydrogen bonded R22(8) motifs of CYT-acetic acid joined in a centrosymmetric arrangement via an R42(8) motif. Hydrogen bonds are shown as dashed lines.

## supplementary crystallographic information

### Comment

Cytenamide (CYT) is an analogue of carbamazepine (CBZ), a dibenzazepine drug used to control seizures (Cyr *et al.*, 1987). CYT-acetic acid solvate was produced during an automated parallel crystallization study (Florence *et al.*, 2006*a*) of CYT as part of a wider investigation that couples automated parallel crystallization with crystal structure prediction methodology to investigate the basic science underlying the solid-state diversity of CBZ (Florence, Johnston, Price *et al.*, 2006*b*; Florence, Leech *et al.*, 2006) and its closely related analogues: CYT, 10,11-dihydrocarbamazepine (DHC) (Bandoli *et al.*, 1992; Harrison *et al.*, 2006; Leech *et al.*, 2007) and cyheptamide (Florence *et al.*, 2008). The sample was identified as a new form using multi-sample foil transmission X-ray powder diffraction analysis (Florence *et al.*, 2003). Subsequent manual recrystallization from a saturated acetic acid solution by slow evaporation at 278 K yielded a sample suitable for single-crystal X-ray diffraction (Fig. 1).

The reported crystal structure is essentially iso-structural with that of CBZ-acetic acid (1/1) (Fleischman *et al.*, 2003) and DHC-acetic acid (1/1) (Johnston *et al.*, 2006). Accordingly, it displays the same space group with very similar unit-cell parameters and packing arrangements [CBZ:acetic *a* = 5.121 (4) Å, *b* = 15.714 (13) Å, *c* = 18.499 (15) Å, β = 95.65 (1)°; DHC:acetic *a* = 5.3104 (4) Å, *b* = 15.424 (17) Å, *c* = 18.7329 (2) Å, β = 95.65 (1)°]. Specifically, the CYT and acetic acid molecules are connected *via* O—H···O and N—H···O hydrogen bonds (contacts 1 and 2) to form an *R*_2_^2^(8) (Etter, 1990) dimer motif. A third hydrogen bond, N1—H1···O2, joins adjacent dimers forming a centrosymmetric double motif arrangement (Fig. 2).

### Experimental

A sample of cytenamide was synthesized according to a modification of the published method (Davis *et al.*, 1964). A single-crystal sample of cytenamide-acetic acid was grown form a saturated acetic acid solution by isothermal solvent evaporation at 278 K.

### Refinement

All non-H atoms were refined anisotropically. H-atoms were found on a difference Fourier map and were initially refined with soft restraints on the bond lengths and angles to regularize their geometry (bond lengths to accepted values, *i.e.* C—H in the range 0.93–98, N—H = 0,86 and O—H = 0.82 Å with esd's of 0.02 Å) and *U*_iso_(H) (in the range 1.2–1.5 times *U*_eq_ of the parent atom), after which the positions were treated with the riding model. Atoms C12, C13, C14 and, to some extent C15, suffer from large and prolate thermal ellipsoids. Given the rigidity of the molecule and well behaved thermal parameters of the remainder atoms, we exclude the possibility of disorder or incorrect treatment of absorption effects. Investigation of diffraction frames indicated significant splitting of some low-order reflections and this is likely to be the principal cause of the anomalous thermal parameters and of high *R*-factor obtained.

### Crystal data

**Table:**

C_16_H_13_NO·C_2_H_4_O_2_	*F*_000_ = 624
*M**_r_* = 295.34	*D*_x_ = 1.279 Mg m^−^^3^
Monoclinic, *P*2_1_/*c*	Mo *K*α radiation λ = 0.71073 Å
Hall symbol: -P 2ybc	Cell parameters from 3006 reflections
*a* = 5.8726 (17) Å	θ = 3–26º
*b* = 14.418 (3) Å	µ = 0.09 mm^−^^1^
*c* = 18.182 (4) Å	*T* = 160 K
β = 95.13 (2)º	Needle, colourless
*V* = 1533.3 (6) Å^3^	0.44 × 0.09 × 0.06 mm
*Z* = 4	

### Data collection

**Table:**

Oxford Diffraction Gemini diffractometer	2759 independent reflections
Monochromator: graphite	2025 reflections with *I* > 2σ(*I*)
Detector resolution: 15.9745 pixels mm^-1^	*R*_int_ = 0.065
*T* = 160 K	θ_max_ = 25.2º
ω scans	θ_min_ = 2.7º
Absorption correction: multi-scan(CrysAlis RED; Oxford Diffraction, 2007)	*h* = −7→7
*T*_min_ = 0.84, *T*_max_ = 0.99	*k* = −17→17
16235 measured reflections	*l* = −21→21

### Refinement

**Table:**

Refinement on *F*^2^	Hydrogen site location: inferred from neighbouring sites
Least-squares matrix: full	H-atom parameters constrained
*R*[*F*^2^ > 2σ(*F*^2^)] = 0.089	Method = Modified Sheldrick *w* = 1/[σ^2^(*F*^2^) + 3.15*P*], where *P* = (max(*F*_o_^2^,0) + 2*F*_c_^2^)/3
*wR*(*F*^2^) = 0.148	(Δ/σ)_max_ = 0.0002
*S* = 1.08	Δρ_max_ = 0.43 e Å^−^^3^
2759 reflections	Δρ_min_ = −0.37 e Å^−^^3^
199 parameters	Extinction correction: None
Primary atom site location: structure-invariant direct methods	

### Fractional atomic coordinates and isotropic or equivalent isotropic displacement parameters (Å^2^)

**Table:**

	*x*	*y*	*z*	*U*_iso_*/*U*_eq_	
C1	0.8743 (7)	0.1556 (2)	0.5586 (2)	0.0377	
C2	1.0683 (7)	0.2065 (3)	0.52488 (19)	0.0394	
H2	1.2072	0.1709	0.5395	0.0464*	
C3	1.0494 (7)	0.2106 (2)	0.4415 (2)	0.0359	
H3	0.3643	0.6194	0.8262	0.0919*	
C4	1.2134 (7)	0.1675 (3)	0.4031 (2)	0.0434	
H4	1.3362	0.1355	0.4291	0.0517*	
C5	1.2009 (8)	0.1694 (3)	0.3270 (2)	0.0489	
H5	1.3155	0.1417	0.3023	0.0588*	
C6	1.0206 (8)	0.2134 (3)	0.2876 (2)	0.0492	
H6	1.0115	0.2146	0.2361	0.0589*	
C7	0.8543 (8)	0.2557 (3)	0.3251 (2)	0.0479	
H7	0.7287	0.2834	0.2980	0.0554*	
C8	0.8695 (7)	0.2579 (2)	0.4025 (2)	0.0391	
C9	0.7017 (8)	0.3120 (3)	0.4383 (2)	0.0500	
H9	0.5540	0.3138	0.4140	0.0595*	
C10	0.7341 (8)	0.3609 (3)	0.5012 (2)	0.0548	
H10	0.6062	0.3933	0.5149	0.0651*	
C11	0.9405 (9)	0.3695 (3)	0.5504 (2)	0.0537	
C12	0.9797 (11)	0.4529 (3)	0.5910 (3)	0.0745	
H12	0.8701	0.4989	0.5867	0.0940*	
C13	1.1748 (15)	0.4673 (4)	0.6361 (3)	0.1040	
H13	1.1957	0.5230	0.6618	0.1120*	
C14	1.3377 (12)	0.3999 (5)	0.6437 (3)	0.0924	
H14	1.4716	0.4096	0.6745	0.1038*	
C15	1.3053 (9)	0.3160 (3)	0.6066 (2)	0.0620	
H15	1.4196	0.2700	0.6121	0.0709*	
C16	1.1082 (8)	0.3006 (3)	0.5609 (2)	0.0448	
C17	0.6177 (7)	0.5488 (3)	0.8287 (2)	0.0425	
C18	0.8000 (7)	0.5266 (3)	0.7809 (2)	0.0554	
H18a	0.9163	0.5730	0.7854	0.0834*	
H18b	0.8713	0.4698	0.7965	0.0835*	
H18c	0.7432	0.5218	0.7303	0.0833*	
O1	0.8339 (5)	0.17284 (18)	0.62261 (13)	0.0490	
O2	0.6154 (5)	0.52248 (18)	0.89214 (14)	0.0484	
O3	0.4556 (5)	0.6020 (2)	0.79594 (15)	0.0614	
N1	0.7638 (6)	0.0891 (2)	0.51942 (16)	0.0425	
H1N	0.7870	0.0813	0.4727	0.0503*	
H2N	0.6669	0.0546	0.5419	0.0502*	

### Atomic displacement parameters (Å^2^)

**Table:**

	*U*^11^	*U*^22^	*U*^33^	*U*^12^	*U*^13^	*U*^23^
C1	0.051 (3)	0.0295 (19)	0.030 (2)	−0.0027 (18)	−0.0055 (18)	0.0048 (16)
C2	0.046 (2)	0.038 (2)	0.032 (2)	−0.0007 (18)	−0.0058 (18)	0.0005 (17)
C3	0.049 (2)	0.0252 (18)	0.033 (2)	−0.0068 (17)	0.0016 (18)	0.0008 (16)
C4	0.056 (3)	0.038 (2)	0.036 (2)	−0.0023 (19)	−0.001 (2)	−0.0003 (18)
C5	0.061 (3)	0.045 (2)	0.041 (2)	−0.002 (2)	0.009 (2)	−0.008 (2)
C6	0.075 (3)	0.044 (2)	0.028 (2)	−0.004 (2)	0.002 (2)	0.0015 (18)
C7	0.064 (3)	0.040 (2)	0.038 (2)	−0.002 (2)	−0.008 (2)	0.0106 (19)
C8	0.052 (3)	0.030 (2)	0.035 (2)	−0.0051 (18)	0.0013 (19)	0.0049 (16)
C9	0.062 (3)	0.038 (2)	0.050 (3)	0.004 (2)	0.006 (2)	0.013 (2)
C10	0.079 (3)	0.030 (2)	0.059 (3)	0.008 (2)	0.029 (3)	0.012 (2)
C11	0.090 (4)	0.038 (2)	0.036 (2)	−0.014 (2)	0.019 (2)	0.0013 (19)
C12	0.141 (5)	0.041 (3)	0.049 (3)	−0.023 (3)	0.046 (3)	−0.005 (2)
C13	0.207 (9)	0.063 (4)	0.049 (3)	−0.076 (5)	0.053 (5)	−0.028 (3)
C14	0.138 (6)	0.100 (5)	0.042 (3)	−0.083 (5)	0.027 (3)	−0.023 (3)
C15	0.082 (4)	0.072 (3)	0.032 (2)	−0.036 (3)	0.007 (2)	−0.009 (2)
C16	0.066 (3)	0.040 (2)	0.029 (2)	−0.017 (2)	0.006 (2)	0.0005 (17)
C17	0.054 (3)	0.034 (2)	0.038 (2)	0.0012 (19)	−0.007 (2)	−0.0052 (18)
C18	0.062 (3)	0.055 (3)	0.048 (3)	0.004 (2)	0.000 (2)	−0.010 (2)
O1	0.073 (2)	0.0466 (16)	0.0272 (15)	−0.0191 (15)	0.0036 (14)	−0.0021 (12)
O2	0.064 (2)	0.0436 (16)	0.0359 (16)	0.0114 (14)	−0.0044 (14)	0.0061 (13)
O3	0.082 (2)	0.067 (2)	0.0351 (16)	0.0330 (18)	0.0035 (15)	0.0077 (14)
N1	0.064 (2)	0.0351 (18)	0.0269 (17)	−0.0113 (16)	−0.0012 (16)	0.0012 (14)

### Geometric parameters (Å, °)

**Table:**

O1—C1	1.234 (4)	C11—C12	1.419 (6)
O2—C17	1.216 (5)	C12—C13	1.364 (10)
O3—C17	1.322 (5)	C13—C14	1.362 (10)
O3—H3	0.8400	C14—C15	1.390 (8)
N1—C1	1.329 (5)	C15—C16	1.381 (6)
N1—H1N	0.8800	C2—H2	0.9800
N1—H2N	0.8800	C4—H4	0.9500
C1—C2	1.529 (6)	C5—H5	0.9300
C2—C16	1.516 (6)	C6—H6	0.9300
C2—C3	1.511 (5)	C7—H7	0.9400
C3—C8	1.397 (5)	C9—H9	0.9400
C3—C4	1.386 (6)	C10—H10	0.9400
C4—C5	1.379 (5)	C12—H12	0.9200
C5—C6	1.379 (6)	C13—H13	0.9300
C6—C7	1.382 (6)	C14—H14	0.9300
C7—C8	1.403 (5)	C15—H15	0.9400
C8—C9	1.455 (6)	C17—C18	1.473 (6)
C9—C10	1.343 (5)	C18—H18A	0.9500
C10—C11	1.446 (6)	C18—H18B	0.9500
C11—C16	1.400 (7)	C18—H18C	0.9500
			
C17—O3—H3	111.00	C1—C2—H2	106.00
H1N—N1—H2N	122.00	C16—C2—H2	105.00
C1—N1—H1N	120.00	C3—C2—H2	106.00
C1—N1—H2N	118.00	C3—C4—H4	120.00
N1—C1—C2	118.5 (3)	C5—C4—H4	119.00
O1—C1—N1	121.7 (3)	C6—C5—H5	120.00
O1—C1—C2	119.7 (3)	C4—C5—H5	120.00
C1—C2—C3	115.5 (3)	C5—C6—H6	120.00
C3—C2—C16	113.2 (3)	C7—C6—H6	120.00
C1—C2—C16	110.4 (3)	C6—C7—H7	119.00
C4—C3—C8	119.4 (3)	C8—C7—H7	120.00
C2—C3—C4	119.7 (3)	C10—C9—H9	116.00
C2—C3—C8	120.9 (3)	C8—C9—H9	116.00
C3—C4—C5	121.3 (4)	C11—C10—H10	116.00
C4—C5—C6	120.0 (4)	C9—C10—H10	116.00
C5—C6—C7	119.4 (3)	C11—C12—H12	119.00
C6—C7—C8	121.4 (4)	C13—C12—H12	119.00
C3—C8—C7	118.4 (4)	C12—C13—H13	120.00
C7—C8—C9	118.4 (4)	C14—C13—H13	120.00
C3—C8—C9	123.1 (3)	C15—C14—H14	120.00
C8—C9—C10	127.8 (4)	C13—C14—H14	120.00
C9—C10—C11	128.3 (4)	C14—C15—H15	120.00
C10—C11—C12	118.9 (4)	C16—C15—H15	120.00
C12—C11—C16	116.8 (4)	O2—C17—C18	124.2 (4)
C10—C11—C16	124.3 (4)	O3—C17—C18	113.1 (3)
C11—C12—C13	122.0 (5)	O2—C17—O3	122.7 (4)
C12—C13—C14	119.8 (5)	C17—C18—H18A	110.00
C13—C14—C15	120.5 (6)	C17—C18—H18B	110.00
C14—C15—C16	120.2 (5)	C17—C18—H18C	112.00
C2—C16—C11	119.8 (4)	H18A—C18—H18B	107.00
C11—C16—C15	120.6 (4)	H18A—C18—H18C	109.00
C2—C16—C15	119.5 (4)	H18B—C18—H18C	109.00
			
O1—C1—C2—C3	−157.1 (3)	C5—C6—C7—C8	2.5 (7)
O1—C1—C2—C16	−27.1 (5)	C6—C7—C8—C3	−4.4 (6)
N1—C1—C2—C3	27.5 (5)	C6—C7—C8—C9	173.5 (4)
N1—C1—C2—C16	157.4 (3)	C3—C8—C9—C10	33.2 (6)
C1—C2—C3—C4	−116.5 (4)	C7—C8—C9—C10	−144.6 (4)
C1—C2—C3—C8	64.0 (4)	C8—C9—C10—C11	−2.4 (7)
C16—C2—C3—C4	114.9 (4)	C9—C10—C11—C12	149.6 (5)
C16—C2—C3—C8	−64.6 (5)	C9—C10—C11—C16	−30.0 (7)
C1—C2—C16—C11	−67.0 (5)	C10—C11—C12—C13	−177.3 (5)
C1—C2—C16—C15	110.0 (4)	C16—C11—C12—C13	2.4 (8)
C3—C2—C16—C11	64.2 (5)	C10—C11—C16—C2	−5.9 (6)
C3—C2—C16—C15	−118.8 (4)	C10—C11—C16—C15	177.2 (4)
C2—C3—C4—C5	179.6 (4)	C12—C11—C16—C2	174.4 (4)
C8—C3—C4—C5	−0.8 (6)	C12—C11—C16—C15	−2.5 (6)
C2—C3—C8—C7	−177.0 (4)	C11—C12—C13—C14	−0.8 (9)
C2—C3—C8—C9	5.2 (5)	C12—C13—C14—C15	−0.9 (9)
C4—C3—C8—C7	3.5 (5)	C13—C14—C15—C16	0.8 (8)
C4—C3—C8—C9	−174.3 (4)	C14—C15—C16—C2	−175.9 (4)
C3—C4—C5—C6	−1.1 (7)	C14—C15—C16—C11	1.0 (7)
C4—C5—C6—C7	0.3 (7)		

### Hydrogen-bond geometry (Å, °)

**Table:**

*D*—H···*A*	*D*—H	H···*A*	*D*···*A*	*D*—H···*A*
N1—H1N···O2^i^	0.88	2.27	2.888 (4)	128
N1—H2N···O2^ii^	0.88	2.18	3.018 (4)	158
O3—H3···O1^iii^	0.84	1.73	2.565 (4)	169

Symmetry codes: (i) *x*, −*y*+1/2, *z*−1/2; (ii) −*x*+1, *y*−1/2, −*z*+3/2; (iii) −*x*+1, *y*+1/2, −*z*+3/2.
